# Association of Tongue Bacterial Flora and Subtypes of Liver-Fire Hyperactivity Syndrome in Hypertensive Patients

**DOI:** 10.1155/2018/9536924

**Published:** 2018-01-10

**Authors:** Jie-wei Luo, Cong-huai Lin, Yao-bin Zhu, Xing-yu Zheng, Yong-xi Wu, Wei-wei Chen, Xiao Yang

**Affiliations:** ^1^Provincial Clinical Medical College, Fujian Medical University, Fuzhou 350001, China; ^2^Department of Traditional Chinese Medicine, Fujian Provincial Hospital, Fuzhou 350001, China; ^3^Department of Traditional Chinese Medicine, The First Affiliated Hospital, Fujian Medical University, Fuzhou 350005, China; ^4^Teaching and Research Office of Medical Cosmetology, Department of Management, Fujian Health College, Fuzhou 350101, China

## Abstract

Structural changes in symbiotic human microorganisms can affect host phenotype. Liver-fire hyperactivity syndrome (LFHS) presents as bitter taste, halitosis, xerostomia, odontalgia, and other oral symptoms. LFHS is associated with hypertension (EH). In this study, tongue flora was analyzed to further understand the intrinsic relationship between tongue flora and LFHS. Samples of tongue coating, from 16 patients with EH-LFHS, 16 with EH-non-LFHS, and 16 controls, were obtained; then, 16S rRNA variable (V3-V4) regions were amplified and sequenced by MiSeq PE300 Sequencing. Tag clustering and Operational Taxonomic Units (OTUs) abundance analysis were used to compare the OTU sequence with the 16S database. The species were classified, and diversity and structure of the bacterial flora were compared between the three groups. Alpha diversity analysis, including Observed Species index and Chao index, indicated significantly higher richness of species in patients with EH-LFHS (*p* < 0.05). Higher phylogenetic diversity, in patients with EH-non-LFHS, indicates greater differences in evolutionary history than in patients with EH-LFHS.* Streptococcus*,* Rothia*,* Neisseria*, and* Sphingomonas* were the most prevalent in patients with EH-LFHS, differed from the other two groups. This indicates that richer bacterial diversity, and structure associated with EH-LFHS, may affect the occurrence, development, and outcome of hypertension and syndrome subtypes recognized by TCM.

## 1. Introduction

The human microbiome refers to the collective genomes of microorganisms (symbiotic and pathogenic) that reside in and on the human body [1]. The microbiome is fundamentally important to the metabolism of the body. Within the past decade, programs to better understand the human microbiome in general, and more specifically the oral microbiome, have included the Human Microbiome Project (HMP) of the United States National Institutes of Health (NIH) [2] and the Human Oral Microbiome Database (HOMD) of the U.S. National Institute of Dental and Craniofacial Research (NIDCR) [3]. HOMD provides a detailed record of the type, metabolism, and pathogenicity of oral bacteria.

Traditional Chinese Medicine (TCM) has been practiced for over 5000 years. TCM relies on syndrome differentiation and treatment. Examination of the tongue is fundamentally important, because the tongue is considered a mirror of visceral changes that can predict an alteration in internal “Yin” and “Yang” of the body. In TCM, examination of the tongue provides an objective assessment of the state of habitus (strong or weak) and the rise and fall of vital Qi, which can be prognostic. The microbial flora, which coats the tongue, is formed by the Qi of the spleen and stomach. The coating on the tongue is normally thin and white, moderately wet, and the center and root of the tongue are a bit thick; this reflects the Qi of the stomach. Pathologies of the stomach can be reflected in the composition and appearance of the tongue flora [4].

The symbiotic microbiota of the human body has been termed “the second genome of the human body.” Microorganisms are presumed to be the cause of variation that can affect the host phenotype [5]. According to TCM, the human and microbial genomes must be in harmonious coexistence to achieve the balance between Yin and Yang. The bacterial flora, of the coating of the tongue, is an important component of the oral flora, which significantly affects human health and is closely related to multisystem diseases.

Liver-fire hyperactivity syndrome (LFHS) presents as bitter taste, halitosis, xerostomia, odontalgia, and other oral symptoms. LFHS has been associated with hypertension [6, 7]. To better understand the intrinsic relationship between the flora of the tongue and liver-fire hyperactivity, we sequenced the oral flora of patients with LFHS, with and without essential hypertension (EH), as well as the flora of healthy individuals. For this, we used the U341F/U806R primer in the V3-V4 region from the variable region of 16sRNA. This approach broadly examines oral bacteria and archaea [8, 9].

## 2. Materials and Methods

### 2.1. Subjects

We recruited 32 patients, who had visited the Fujian Provincial Hospital from December 2014 to December 2015 and met the EH inclusion/exclusion criteria detailed below. These included 16 patients with EH-liver-fire hyperactivity syndrome (EH-LFHS; 8 men and 8 women; average age 69.94 ± 8.84 years) and 16 patients with EH-non-liver-fire hyperactivity syndrome (EH-non-LFHS; 9 men and 7 women; average age 66.63 ± 11.98 years). Sixteen healthy control subjects, from the same hospital medical center, during the same period, were also enrolled (7 men and 9 women; average age 63.38 ± 8.40 years). The age and gender ratio of the three groups were comparable (both *p* > 0.1).

### 2.2. Inclusion and Exclusion Criteria

All subjects were patients with hypertension without other significant diseases. The diagnostic criteria for hypertension were from the 2010 Chinese Guidelines for the Management of Hypertension [10]. Diagnostic criteria for liver-fire hyperactivity and EH-LFHS diagnostic criteria have been previously detailed [7]. The EH-LFHS diagnostic criteria included the main symptoms of dizziness, headache, and irritability. Secondary symptoms included flushing; ocular redness; dry mouth; bitter taste; constipation; dark urine with burning sensation; reddened tongue with thin and yellow fur; and wiry, rapid pulse. The exclusion criteria comprised presence of major diseases such as diabetes, coronary heart disease, heart failure, liver and kidney dysfunction, lung disease, tumor, stroke, or infection; pregnancy or lactation in women; smoking; and drinking.

### 2.3. Sample Acquisition and DNA Extraction

Samples of tongue coating were acquired as described in Version 12.0 of the procedure manual for the Human Microbiome Project (Accession: phd003190.2). Alcohol consumption and gargling were not allowed for the day preceding sampling. Patients fasted for 8 hours prior to sampling and did not brush their teeth on the day of sampling. For sampling, a 1 cm^2^ area at the center of the tongue was swabbed for 5 seconds using a Catch-All™ sample collection swab (Lucigen Corporation, Middleton, WI, USA). Immediately after swabbing, the swab was swirled in 750 *μ*L of MoBio buffer in a MoBio tube. The swab sponge was pressed against the tube wall, multiple times for 20 seconds, to ensure the transfer of bacteria from the swab to the solution. After 30 minutes, the tube was transferred to an −80 centigrade freezer and stored until analysis.

For analysis, DNA was extracted, using a PowerSoil DNA isolation kit (Qiagen, Valencia, CA, USA), according to the manufacturer's instructions. PCR was conducted using a 16S rRNA universal primer 27F/1492R (27F: 5′-AGAGTTTGATCMTGGCTCAG-3′; 1492R: 5′-TACGGYTACCTTGTTACGACTT-3′; Thermo Fisher Scientific, Waltham, MA, USA).

### 2.4. Sequencing of the V3-V4 Region and 16S rRNA

16S specific primers were used to amplify the V3-V4 variable region. Amplification fragments of 425 bp were obtained. Adaptor linkage was applied, and sequencing was conducted using a MiSeq PE300 apparatus (Illumina, San Diego, CA, USA). Paired-end reads, of 300 bp, were concatenated into longer tags using PANDAseq software as described previously [11]. Tag quality control included the following criteria: (1) no more than three ambiguous Ns on a tag; (2) restricted length between 300 and 500 bp; and (3) average base quality higher than Q20 (Kemp and Aller, 2004). Tags failing to satisfy these three requirements were discarded. Primers for sequencing bacterial 16S rDNA were as follows: U341: CCTACGGGRSGCAGCAG; U806: GGACTACVVGGGTATCTAATC [8, 9]. Sequencing was conducted by Realbio Genomics Institute (Shanghai, China).

### 2.5. Tag Clustering and Operational Taxonomic Unit (OTU) Abundance

Tag sequences were checked for copy number. Tags, in which the sequences appeared at least twice, were considered reliable and were replicated. The replicated sequences were gathered into OTUs based on a software-assessed similarity of 0.97, and OTU-representing tags were chosen. All the tags of high quality were mapped on OTU-representing tags, with same similarity, to get the real abundance for each OTU. An OTU-to-sample abundance table was generated [12]. The database of the Ribosomal Database Project (RDP) was used to compare representative sequences with the 16S DNA of known species, and species classification was conducted for each OTU [13].

### 2.6. Diversity Analysis

Alpha diversity was conducted using Qiime python scripts. The purpose of alpha diversity is to determine the lowest number of tags necessary to achieve a plateau of rarefaction curves; therefore, all the tags were used. In the case of beta diversity, in which comparisons were made between samples, the tags of each sample were randomly downsized to the same number, even though we used relative abundances. Analyses were conducted using R statistical software (R Version 3.1.3).

### 2.7. Ethics Statement

This study was performed at the Fujian Provincial Hospital with the approval of the Medical Ethics Committee (K2014-005-01) of this hospital. Written informed consent was obtained from each patient.

## 3. Results

### 3.1. Alpha Diversity and Drainage Analyses

**Figure 1:**
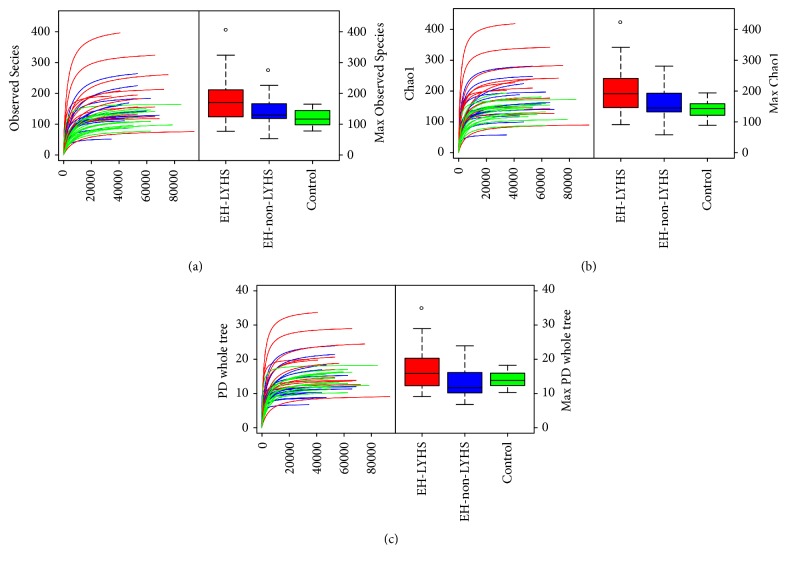
Curves of the Observed Species index (a), Chao index (b), and PD whole tree index (c). In (a) and (b), the horizontal axis represents the number of clean reads randomly extracted from a sample, and the vertical axis indicates the alpha diversity index corresponding to the number of reads. Alpha diversity reflects the diversity of species in single samples including the Observed Species index, Chao index, and phylogenetic diversity (PD) whole tree index. Observed Species and Chao index reveal the richness of a community of species in the samples, regardless of the abundance of each species in the community. (c) The difference in the evolutionary history of species in a sample. The larger the PD whole tree index, the greater the difference of the species preserved in evolutionary history. A curve in the graph represents a sample. Outliers are marked with “∘.”

Alpha diversity reflects the diversity of the species in individual samples including Observed Species index, Chao index, and phylogenetic diversity (PD) whole tree index [14]. Species richness data, of the Observed Species and Chao indices, revealed significantly greater richness in the EH-LFHS group than in the other two groups (*p* < 0.05). The PD whole tree index reflects the evolutionary history of the sample species. Compared with the EH-non-LFHS samples, the larger PD whole tree of the EH-LFHS samples (Figure 1 and Table 1) indicated a greater remaining difference of evolutionary history.

### 3.2. OTU

**Figure 2:**
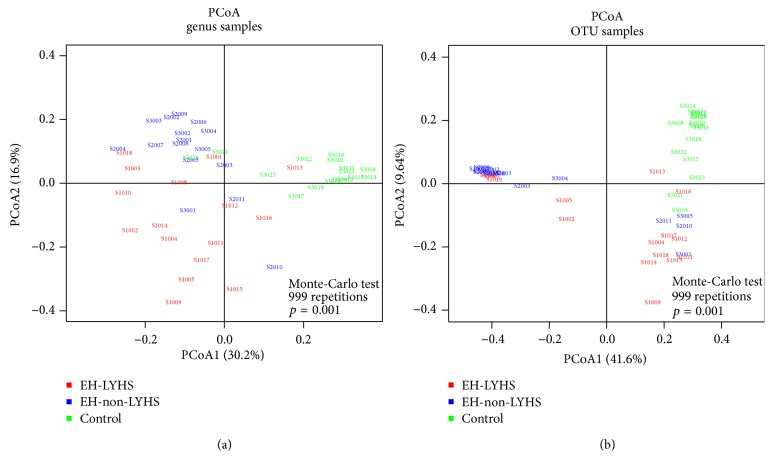
Principal Coordinates Analysis (PCoA) charts of OTU and genus samples. PCoA analysis reflects a dimensionality reduction and is a technique for analyzing and simplifying data sets. If the two samples are closer in distance, the compositions of the two samples will be more similar.

OTU abundance initially explained the abundance of species in the samples. The Kruskal test indicated that the clean reads, mapped reads, and final OTU of the EH-LFHS group were significantly higher than those in the other two groups (all *p* < 0.05; Table 2). Most of the samples appeared to be well sequenced, with 30000 or 34800 tags randomly picked for each sample. By contrast, the EH-LFHS group was most diverse and had more OTUs. As compared with the EH-non-LFHS group, the EH-LFHS group had more OTUs in common with the healthy controls. In this experiment, 992 different OTUs were obtained, and there were 231 significant OTU differences among the three groups (*p* < 0.05). The Principal Coordinates Analysis (PCoA), of the OTU abundance in each sample, indicated that there were significant differences in OTU abundance among the groups (*p* < 0.05) and that the three groups were well separated, according to OTU type, upon mapping (Figure 2(a)).

### 3.3. Analysis of Sample Complexity and Significant Differences

**Figure 3:**
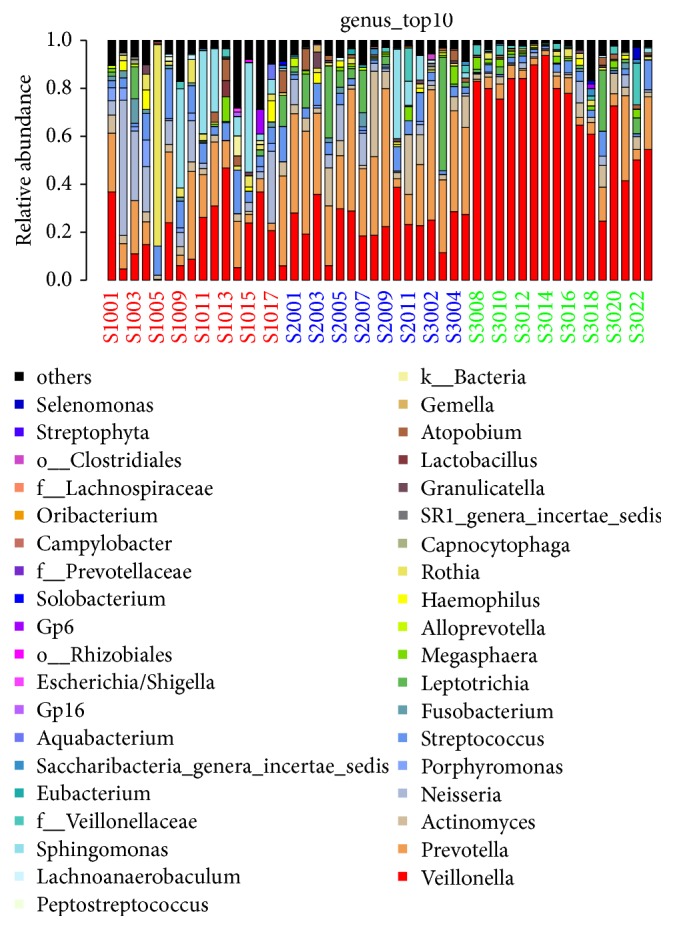
The profiling histogram of the former top 10 species in each sample at the genus level.

**Figure 4:**
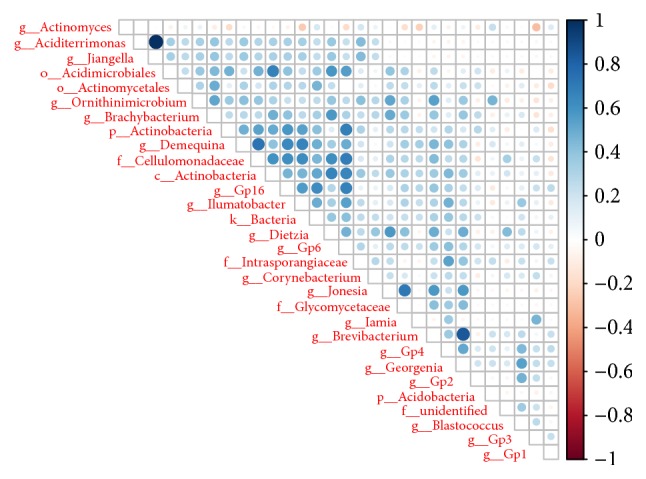
Symbiosis and antagonism of various fungi at the genus level. At the genus level, the strength of symbiotic and antagonistic relationships was obtained by rank sum test, and there was a correlation among the top 30 differentiated species. The blue on the right shows a positive correlation, and the red shows a negative correlation. The darker the color, the stronger the correlation between the species.

The species-profiling histogram can directly determine the specific species and their abundance, in a sample. The profiling histograms of the former top 10 species in each sample, at the genus level, are shown in Figure 3. The relationships among species are mainly symbiotic and antagonistic, and interspecific relationships can affect the abundance of the species.  Figure 4 shows the strength of symbiotic and antagonistic relationships among the various genera. PCoA analysis indicated that samples were separated by groups at the genus level (Figure 2(b)). This suggests that disease status was the main factor in the significantly different bacterial composition (*p* < 0.05). Hence, it is likely that the diversity of microorganisms coating the tongue was strongly related to EH-LFHS and EH-non-LFHS.

### 3.4. Linear Discriminant Analysis (LDA)

**Figure 5:**
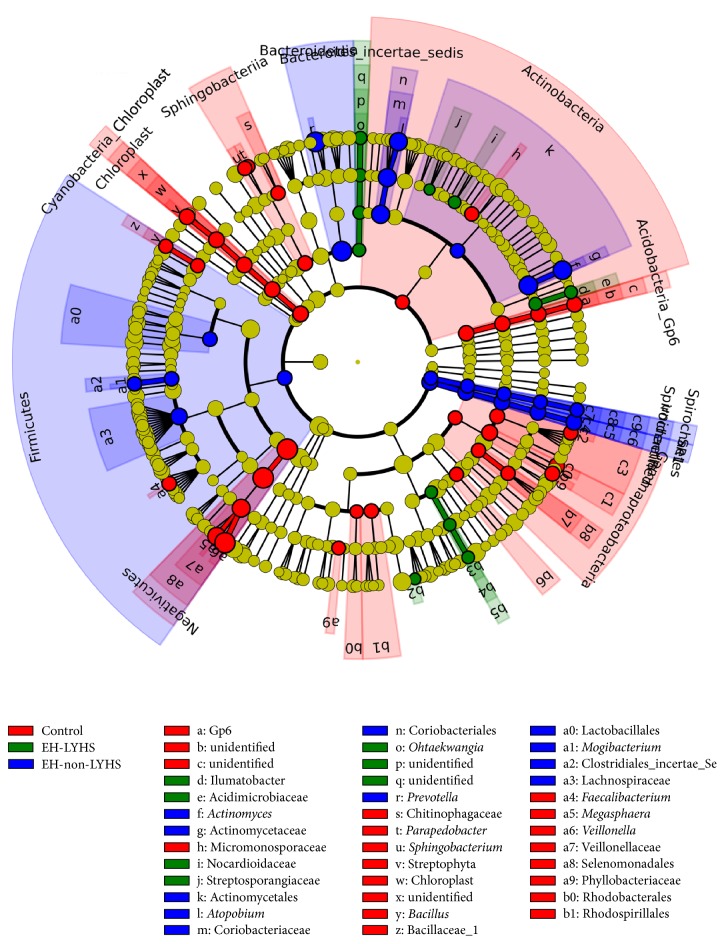
Evolutionary bifurcation diagram of LDA effect size. The evolutionary bifurcation diagram can visually represent the major species, indicating the differences in each group; inner to outer circles represent the taxonomic level from the phylum to the genus. Each small circle, at different classification levels, represents a classification under this level; the diameter of the small circle is proportional to relative abundance.

The LDA effect size (LEfSe) tools are used to estimate how the abundance of each component (species) affects the differences between classes or groups; these tools are also used to identify communities or species having significant differences in sample classification [15]. The LDA distribution histograms of each group are presented in Table 3. The length of the histogram represents the extent of the influence of significant difference species. The evolutionary cladogram, shown in Figure 5, visually represents the major species, which result in differences among each group. The results indicate the different bacterial composition between the groups at the genus level. Samples, acquired from healthy controls, were abundant in* Veillonella*, while* Prevotella* was prevalent in the EH-non-LFHS group. The compositions of the samples, acquired from the EH-non-LFHS and control groups, were similar, while that of the EH-LFHS group was relatively more varied. At the phylum level, the difference between the EH-LFHS and EH-non-LFHS groups mainly involved* Proteobacteria* and* Fusobacteria*; EH-LFHS samples contained more* Proteobacteria*. The prevalence of* Firmicutes*,* Bacteroidetes*, and* Actinobacteria* was similar. In the healthy controls,* Firmicutes* predominated. The three groups differed significantly in 54 genera. In addition to* Veillonella* and* Prevotella* (Table 3),* Actinomyces* was most abundant in the EH-non-LFHS group. The prevalence of* Porphyromonas*,* Rothia*,* Parvimonas*, and* Streptococcus* was greater in the EH-LYHS group.* Megasphaera* was somewhat more prevalent in healthy controls.

## 4. Discussion

Disruption of microbial homeostasis can produce clinical symptoms. Microbial stability is correlated with the abundance of relevant tissues. For example, irritable bowel syndrome and inflammatory bowel disease are closely related to the amount of intestinal* Bifidobacterium* [16, 17]. Host genetics are also important; for example, African American women are more susceptible to bacterial vaginosis than non-Hispanic white women [18]. Overuse of antibiotics may cause the translocation of intestinal bacteria and disrupt the stability of microbial communities [19]. Diet is another factor that affects the stability of human microbes. For example, the abundance of gingival anaerobic bacteria decreases more in people who consume coffee and wine than in those who do not; these differences in the microbial population can affect oral health [20].

How human symbiotic bacteria cause disease remains unclear. An imbalance in the intestinal flora may result in metabolic syndrome, peptic ulcer, heart disease, and obesity. Dysregulation of microbial molecules may directly affect the permeability of the intestinal epithelium; via the pituitary adrenal axis (HPA axis), it may also induce inflammatory responses and insulin resistance [21, 22]. In addition, intestinal microflora, such as segmental filamentous bacteria,* Bacteroides*, and spindle cells, can promote the differentiation of T cells, resulting in strong innate and adaptive immune responses in the intestine [23]. Intestinal microflora may affect mood and behavior through a complex brain-gut axis; for example, decreased numbers of intestinal* Clostridium* species have been linked to impaired stress response in mice [24]. Recent research on plasma oxidation of trimethylamine N-oxide (TMAO) has shown that degradation of TMAO can decrease the level of cholesterol in the blood, increasing the risk of atherosclerosis, coronary heart disease, heart failure, and other cardiovascular disorders [25, 26]. Foods, rich in choline or trimethylamine, are the basis of TMAO, which is generated by the action of intestinal microbial degradative enzymes that can rapidly enter the liver through blood. The reaction also involves flavin monooxygenases. TMAO is eventually removed via the kidneys [27, 28]. Although these studies examined the pathogenicity of intestinal microbial disorders, it is possible that metabolites from oral flora can exert similar effects.

Accumulating evidence indicates that oral microbiota is related to periodontal, cardiovascular, and other diseases. An imbalance in microecology may initiate or promote the process of atherosclerosis [29, 30]. Murine macrophages have been cocultured with* P. gingivalis* in the presence of low-density lipoproteins (LDL); this has been shown to promote LDL mobility and lead to dose-dependent formation of foam cells, caused in part by LDL aggregation via proteolysis of ApoB-100 [31]. The formation of foam cells is widely recognized as the initial critical step in atherosclerosis and atherosclerosis markers. Studies using heterozygous apolipoprotein E-deficient [ApoE(+/−)] mice, fed a high-fat diet or regular chow and inoculated or not inoculated with* P. gingivalis*, have shown the following. Proximal aortic lesion size, at 24 weeks, was 9-fold greater in the chow-fed mice inoculated with* P. gingivalis* than in noninoculated mice and was 2-fold greater in* P. gingivalis*-inoculated versus noninoculated high-fat diet-fed mice. In another study, atherosclerotic plaques were rich in macrophages, and* P. gingivalis* ribosomal DNA was found in the aorta, heart, and liver [32]. Oxidation of LDL is mediated by external factors; the above studies suggest that* P. gingivalis* can induce LDL oxidation, enhance vascular oxidative stress, and promote the development of atherosclerosis. Do other oral bacteria have the same effect? Periodontal bacteria have been detected in infected heart valves and aortic aneurysms [33–35].* Chryseomonas*, Wei Rong* Staphylococcus*, and* Streptococcus* were found more prevalent in the oral flora of 15 people with atherosclerosis than in that of healthy individuals; bacterial DNA was also found in the atherosclerotic plaques of the individuals with atherosclerosis [36]. The abundance of Wei Rong* Staphylococcus* and* Streptococcus* in plaque may be related to the abundance of oral white blood cells and the level of plasma cholesterol.

Intestinal flora may be an important environmental factor in hypertension [37]. An imbalance in oral flora may also affect the regulation of blood pressure. In one study, 15 hypertensive volunteers were randomly allocated to the experimental group and control group. The experimental group rinsed their mouths with an antiseptic mouthwash, while the control group used sterile water. After 3 consecutive days, the average systolic blood pressure of the experimental group increased by 2.3 mmHg, while the diastolic blood pressure did not change; oral nitrate reduction in the experimental group was decreased. The levels of nitrite in saliva decreased significantly, while those of nitrate increased; plasma nitrite, in the experimental group, had also decreased [38].

Nitric oxide (NO) is a lipophilic gas. It is a protective factor in the cardiovascular system and plays a key role in maintaining vascular tone and blood pressure. There are two main sources of intravascular NO. Using L-arginine as substrate, NADPH and L-arginine are oxidized to L-citrulline and NO nicotinamide adenine dinucleotide phosphate (NADP) by the action of nitric oxide synthase (NOS). In addition, dietary nitrate is decomposed to nitrite and NO with the help of oral microorganisms, suggesting that oral microorganisms can affect cardiovascular disease in a variety of ways.

In our study, the abundance of* Veillonella* in the tongue coating of healthy controls was higher than that in patients with hypertension. In normal flora,* Veillonella* in the human natural cavity tract is involved with the formation of plaque biofilm and can reduce dental caries.* Streptococcus*, the predominant bacterium associated with EH-LFHS, is a gram-positive bacterium, closely related to oral symptoms such as halitosis.* Rothia* are a gram-positive bacilli that can reduce nitrate.* Neisseria* are gram-negative cocci that are involved in the formation of plaque biofilms. Normal oral parasitic bacteria, such as* Rothia* and* Neisseria*, may be conditional pathogens in endocarditis [39, 40].* Sphingomonas* is gram-negative bacterium, which can convert pentose, hexose, and disaccharide into acids. There are few studies on it thus far.* Prevotella* and* Bacteroides*, present in the normal flora of the oral, gut, and female genital tract, predominated in the EH-non-LFHS group.* Bacteroides* is not highly pathogenic, and its abundance may also be related to oral symptoms.

Fifty-four bacterial genera differed between the three experimental groups, illustrating that differences in the composition of oral flora may be related to the absence or presence of hypertension. This suggests an association between the incidence of hypertension disorders and imbalance in the oral microbiome. The abundance of* Porphyromonas*,* Rothia*,* Parvimonas*,* Streptococcus*, and other bacteria, in the tongue coating of patients with EH-LFHS exhibiting symptoms such as halitosis and bitter taste, was significantly higher than that of the EH-non-LFHS and control groups. This indicates the presence of different flora in TCM syndromes. Volatile sulfide metabolites are produced by the decomposition of sulfur-containing amino acids by oral microorganisms. These metabolites include sulfurated hydrogen, methyl mercaptan, dimethyl mercaptan, benzopyrrole, ammonia, butyric acid, isovaleric acid, and other odorous gases. The biological diversity of the dorsal tongue flora, in patients with halitosis, is higher than that in individuals without halitosis. Leaf* Bacillus*,* Veillonella*,* Solobacterium*, and* Moorei *may be related to halitosis [41]. Increased proportion of oral anaerobic bacteria leads to the release of sulfur protein metabolites, in turn leading to halitosis and other oral symptoms [42, 43]. The change in oral microflora may also affect cholesterol and bile acid metabolism in patients with hypertension, promoting the production of inflammatory factors and endothelial function damage; this plays a role in the pathogenesis of hypertension. Autonomic nerve dysfunction and internal environment disorders, caused by the disease itself, may also cause imbalances in oral flora, worsening the condition.

Therefore, applying TCM to examine the effects of the oral environment can be instrumental in the prevention and treatment of hypertension and the balance of Yin and Yang.

## Figures and Tables

**Table 1:** Statistical results of alpha diversity of the groups.

Group	Chao index	Observed Species index	PD whole tree index
Liver-fire hyperactivity group	191.5 (144.75–241.5)	170.5 (123.5–212.75)	16.5 (12.25–20.75)
Non-liver-fire hyperactivity group	145 (130.25–195)	129.5 (117.75–168.25)	12.5 (10.25–16.75)
Healthy control group	143.5 (119.75–159.5)	116.5 (97.5–145.75)	14.5 (13–16.75)
Chi square value	7.561	7.839	5.003
*p* value	0.023	0.02	0.082^a^

^a^Liver-fire hyperactivity group versus non-liver-fire hyperactivity group; *p* < 0.05.

**Table 2:** Statistical results of clean reads, mapped reads, and final OTU of each group.

Group	Clean reads	Mapped reads	Final OTU
Liver-fire hyperactivity group	83956 (69233–105696)	57093 (44645–69015)	163 (121–204)
Non-liver-fire hyperactivity group	70694 (60496–89226)	48645 (41072–63836)	123 (114–159)
Healthy control group	78274 (72718–90787)	57515 (49859–65527)	114 (87–136)
Chi square value	2.576	3.113	8.824
*p* value	0.276	0.211	0.012

**Table 3:** Species with significant differences in abundance in EH-non-LYHS, EH-non-LYHS, and control group, as assessed by Linear Discriminant Analysis (LDA).

LDA score (log 10)	Liver-fire hyperactivity group	Non-liver-fire hyperactivity group	Healthy control group
8 < score ⩽ 10		Bacteroidia*>Prevotella> *Actinomycetaceae*> Actinomyces*	Selenomonadales*>*Negativicutes*>Veillonella*

6 < score ⩽ 8	Streptosporangiaceae*> Achromobacter> * Nocardioidaceae*> * unidentified*> * Methylophilales*> * Methylophilaceae*> * *Methylophilus> * unidentified*> * *Ilumatobacter> * Acidimicrobiaceae*> * *cteroidetes_incertae_*sedis*> Ohtaekwangia*	Coriobacteriales*> *Coriobacteriaceae*> * *Atopobium> * Lachnospiraceae*> * SRl_genera_incertae_sedis *>SRl*>unidentified> unidentified>unidentified> *Mogibacterium*> *ridiales_lncertae_*Sedis_XI 11> *Actinomycetales*	Veillonellaceae*>Megasphaera>*Gammaproteobacteria*>*Phyllobacteriaceae*> Actinobacteria>Sphingobacterium>*Myxococcales*>*Rhodobacterales*> * *Parapedobacter>*Micromonosporaceae*> * *Stenotrophomonas>*Pseudomonadales*> * *Faecalibacterium>*Rhodospirillales*> * *Acinetobacter>*Bacillaceae_1*>Bacillus> * *Pseudomonas>*Cyanobacteria*_*Chloroplast*> * unidentified*>*Acidobacteria*_*Gp6*>*Gp6*> * unidentified*>*unidentified*>*Streptophyta*> * *Chloroplast>Chloroplast>*Sphingobacteriia*> * Xanthomonadales*>*Chitinophagaceae

4 < score ⩽ 6		Firmicutes*>*Lactobacillales*> Treponema>*Spirochaetia *>*Spirochaetes*> * Spirochaetaceae*> * Spirochaetales	Enterobacteriales*>*Enterobacteriaceae

*Note*. LDA score (log⁡10) represents the magnitude of the effect in species with significant differences in abundance.

## Abbreviations

TCM: Traditional Chinese Medicine
EH: Essential hypertension
EH-LFHS: EH-liver-fire hyperactivity syndrome
EH-non-LFHS: EH-non-liver-fire hyperactivity syndrome
LFHS: Liver-fire hyperactivity syndrome
OTUs: Operational Taxonomic Units
TMAO: Trimethylamine N-oxide
TMA: Trimethylamine
FMOs: Flavin monooxygenase
LDL: Low-density lipoprotein
NO: Nitric oxide
NOS: Nitric oxide synthase
NADP: Nicotinamide adenine dinucleotide phosphate.
